# Clinical outcomes in post-epikeratophakic eyes after removal of epikeratoplasty lenticule

**DOI:** 10.1186/s12886-021-02109-9

**Published:** 2021-09-29

**Authors:** Young-ho Jung, Mee Kum Kim

**Affiliations:** 1 Department of Ophthalmology, Seoul National University College of Medicine, 103 Daehak-ro, Jongno-gu, Seoul, 03080 Republic of Korea; 2 Department of Ophthalmology, Seoul National University Hospital, 101 Daehak-ro, Jongno-gu, Seoul, 03080 Republic of Korea; 3 Laboratory of Ocular Regenerative Medicine and Immunology, Biomedical Research Institute, Seoul National University Hospital, 101 Daehak-ro, Jongno-gu, Seoul, 03080 Republic of Korea

**Keywords:** Epikeratophakia, Reversiblity, Complication, Epikeratophakic lenticule

## Abstract

**Background:**

Assessment of the optical outcome and adverse events in post-epikeratopathic eyes after removal of the epikeratoplasty lenticule (EKPL).

**Methods:**

This was a retrospective case-series study of patients who underwent EKPL removal between 2002 and 2020. Ten eyes were included in the analysis. We compared the clinical characteristics of the patients before surgery, 6 months after surgery, before lenticular removal, and after removal, and reported optical or ocular surface complications.

**Results:**

We removed EKPL due to the lenticular opacity in five eyes (50%), intraocular lens (IOL) insertion (*n* = 4, 40%) after cataract surgery (*n* = 3) or in aphakic eyes (*n* = 1), and lenticule-induced irregular astigmatism in one eye (10%). After EKPL removal, the mean refractive power of the cornea (Km) revealed a tendency to increase. Out of nine cases, six cases showed corneal steepening and three cases revealed corneal flattening. When the keratometric readings of pre-epikeratoplasty and post-lenticular removal were compared within the same case, the average difference was 5.1 D ± 4.0 (*n* = 8). Complications were observed in 3 of 10 cases (excessive corneal flatness, ectatic change, and abnormal epithelial cell ingrowth) after removal.

**Conclusions:**

The surgeon should expect the corneal refractive power to steepen or flatten in some cases with abnormal astigmatism and irregularity. Epikeratophakic eyes may exhibit serious ectatic changes, and abnormal epithelial cell ingrowth after removal of epikeratophakic lenticules.

## Background

Epikeratophakia (EKP) was first described about 40 years ago [1]. Since then, it has been used as a refractive surgical procedure to correct the large refractive errors associated with aphakia, high myopia, and keratoconus [1–6]. EKP uses a corneal lenticule to alter corneal curvature. The procedure is performed with a lamellar disk from a donor cornea or a commercial corneal lenticule that has been optically modified and is then transplanted onto the anterior surface of the cornea. However, due to its varying visual outcome, it has been replaced with newer technologies such as photorefractive keratectomy (PRK), laser in situ keratomileusis (LASIK), laser epithelial keratomileusis (LASEK), and small incision lenticule extraction (SMILE) [7].

Recently, patients who had received EKP in the past have revisited with ocular disease. Among these patients, lenticular removal is required for cataract surgery or lenticular opacity. In the past, there have been studies that published long-term results of EKP [8], but there have been few studies about the clinical outcomes after lenticular removal. To the best of our knowledge, all published studies are case reports, including a few cases [8–10]. To inform the patients what happens next after removal of a lenticule before the surgery, we should know the possible side effects of the epikeratophakic lenticule (EKPL) removal procedure. Therefore, we assessed the optical outcomes and adverse events in post-epikeratophakic eyes after removal of the epikeratoplasty lenticule.

## Methods

This study was approved by the Institutional Review Board of Seoul National University Hospital (IRB No. 2103–080-1204, Seoul, Republic of Korea) and adhered to the Declaration of Helsinki. Informed consent was waived by the IRB because the study was based on a retrospective review of old charts. This was a retrospective case-series study of patients who underwent EKPL removal between 2002 and 2020 at Seoul National University Hospital (a tertiary referral center) in the Republic of Korea. The following data were collected from the medical chart review: information on demographic outcomes, general medical and ocular history, ocular biometric characteristics, and adverse events.

A total of 12 patients and 16 eyes underwent surgery at our hospital. All EKPs were performed between 1991 and 1992. Cases were excluded if the duration of follow-up was less than 3 months, or if the keratometric data had not been measured. Of the 16 patients, three were excluded due to lack of follow-up, and another three were excluded due to the absence of keratometric values.

The EKPL was removed uneventfully. Dissection of the 8 mm optical zone (mid-peripheral cornea) using a Sinskey hook permitted access to the peripheral wing of the epikeratophakic button, which was lifted off easily, leaving a smooth Bowman layer intact over the central cornea. A 1 mm wide trench was left at the 8 mm optical zone and closed naturally with fibrosing wound healing, leaving an annular faint scar.

In the 1990s, before EKP and 6 months after EKP, all patients underwent an ophthalmic examination including corneal K value, refractive error measurements using an auto-kerato-refractometer (Atlas; Carl Zeiss Meditec, Dublin, CA), and manual refraction. Topography was not available at the time.

Since the 2000s, when patients came to remove the lenticule, ocular biometric parameters including K value, refractive errors, and corneal thickness were measured using an auto-kerato-refractometer (Atlas, Carl Zeiss Meditec, Dublin, CA), topography (Orbscan II Bausch & Lomb, Claremont, CA), and ultrasound (US) pachymetry (Axis II PR; Quantel Medical, Bozeman, MT).

We compared the clinical characteristics of the patients before surgery, 6 months after surgery, before lenticular removal, and after the removal procedure, and reported optical or ocular surface complications.

## Result

### Demographics and clinical characteristics of cases that underwent epikeratoplasty

Demographic and clinical features are shown in Table 1. A total of 10 cases were available. Four female (40%) and six male (60%) patients were analyzed. Mean age at EKP was 24.2 years ±10.6 years (range, 5 years – 48 years). Nine patients received EKP for correction of high myopia, and only one patient underwent EKP for correction of traumatic aphakia. The mean age was 42.9 ± 12.7 years (range, 18 ~ 66 years) when the cases underwent removal procedure. The average period after EKP was 18.7 years ±5.0 years (range, 12 years – 30 years).

**Table 1:** Demographic and clinical characteristics of 10 cases

No.	Sex/Age^a^	Laterality	Op date	Diagnosis	Removal date	Reason for Removal
1	M/21	Left	1992.07	High myopia	2004.08	Graft opacity
2	F/21	Right	1990.02	High myopia	2005.05	Cataract surgery
3	F/21	Left	1992.12	High myopia	2004.05	Irregular astigmatism
4	F/5	Left	1992.10	Aphakia	2005.05	IOL insertion
5	M/48	Left	1991.12	High myopia	2009.11	Graft opacity
6	F/22	Right	1991	High myopia	2012.01	Graft opacity
7	M/20	Left	1991.04	High myopia	2012.02	Graft opacity
8	M/32	Right	1991	High myopia	2013.05	Cataract surgery
9	M/32	Left	1991	High myopia	2012.11	Cataract surgery
10	M/20	Right	1991.01	High myopia	2020.03	Graft opacity

*M* Male, *F* Female; ^a^age at the time of EKP surgery

We removed epikeratophakic lenticules (EKPL) due to lenticular opacity in five eyes (50%), intraocular lens (IOL) insertion (*n* = 4, 40%) after cataract surgery (*n* = 3), or in aphakic eyes (*n* = 1), and lenticule-induced irregular astigmatism in one eye (10%).

### Long-term outcome of epikeratophakia and biometric parametric changes after removal of lenticule

Best corrected visual acuity (BCVA) and ocular biometric parameters obtained by an auto-kerato-refractometer (Atlas; Carl Zeiss Meditec, Dublin, CA) are revealed in Table 2.

**Table 2:** Long term outcome of epikeratophakia and clinical feature after removal of lenticule

No	BCVA				Refraction (D)				Keratometry (D)			
	Pre-EK	Post-EK	BR	PR	Pre-EK	Post-EK	BR	PR	Pre-EK	Post-EK	BR	PR
1	20/40	20/66	20/66	20/66	−30.0 -1.5180	−10.0 -10.0 90	N/A	N/A	42.25 / 44.75	N/A	39.62 / 33.62	43.62 / 46.00
2	20/22	20/20	20/66	20/28	−16.0 -2.0180	+ 0.5–2.75180	−9.00 -6.00 5	−22.50 -5.75 13	44.18 / 46.25	N/A	38.14 / 41.82	48.6 / 46.8^a^
3	20/20	20/50	20/2000	N/A	−12.0 -1.5150	− 1.0 -7.25180	N/A	N/A	43.87 / 45.25	46.1 / 38.7	N/A	48.4 / 47.4^a^
4	20/400	20/66	20/400	20/4000	+ 10.5–1.0 90	+ 7.0–1.5180	+ 7.5–3.5180	+ 2.00–6.00180	45.56 / 41.12	N/A	43.50 / 48.37	37.6 / 28.7^a^
5	20/100	20/100	20/1000	20/2000	−31.0 -1.0 90	+ 2.00–1.50 90	N/A	N/A	46.0 / 46.12	N/A	55.2 / 38.0^a^	52.75 / 48.75
6	20/22	N/A	20/22	N/A	−22.0 -3.0180	N/A	−16.75 -2.0 85	N/A	46.25 / 45.63	N/A	43.0 / 41.75	50.0 / 48.3
7	20/22	20/22	20/200	20/1000	−12.75 -2.5180	−6.25 -0.50 90	−18.0 -7.0107	N/A	45.0 /43.75	39.5 / 38.75	53.50 / 48.00	59.25 / 55.75
8	N/A	N/A	20/133	20/66	N/A	N/A	−15.25 -3.25 45	−22.75 -3.50171	N/A	N/A	46.5 / 44.1^a^	45.50 / 44.25
9	N/A	N/A	20/100	20/200	N/A	N/A	−14.75 -2.25 10	−24.50 -5.75175	N/A	N/A	40.8 / 39.7	45.50 / 44.00
10	20/25	20/22	20/40	20/100	−17.0 -5.0180	−0.5 -3.00180	−23.50 -8.25100	N/A	41.87 / 45.25	41.75 / 48.50	47.50 / 45.75	47.25 / 44.00

*BCVA* Best corrected visual acuity, *Pre-EK* Pre-epikeratoplasty, *Post-EK* Post—epikeratoplasty, *BR* Before lenticule removal, *PR* Post lenticule removal, *N/A* Not-available data due to over-ranged values, severe irregular astigmatism, or ocular media opacities in keratometry or refraction; ^a^ Keratometric data were replaced with topography value when the keratometric data were not available

The mean BCVA was 20/30 ± 20/57 (range, 20/400–20/20, *n* = 8) preoperatively. Six months after EKP, it was maintained at 20/33 ± 20/57 (range, 20/100–20/20, *n* = 7). However, it decreased to 20/80 ± 20/800 (range 20/100–20/40, *n* = 10) at pre-EKPL removal and did not improve after EKPL removal (20/100 ± 20/100, range 20/1000–20/28, *n* = 8).

The mean of spherical equivalent refraction (SE) of high myopia cases was − 21.4 D ± 7.0 (range, − 12.75 – -30.75 D, *n* = 7) preoperatively, and − 4.64 D ± 5.26 (range, − 15.0 – + 1.25 D, *n* = 6) postoperatively. The average corrected refractive error quantities were 16.3 D ± 8.3. In the aphakia case, the preoperative manifest refraction was + 10.0–1.00 × 90, and postoperative manifest refraction was + 7.0–1.5 × 180.

Compared with Post-EKP (mean SE, − 3.2 D – ± 2.4, *n* = 3), excessive myopic shift was observed at pre-EKPL removal (mean SE, − 20.4 D ± 6.4, n = 3). Even the mean SE of the pre-EKPL removal state was more myopic than the preoperative mean SE (− 16.8 D ± 2.3, n = 3).

After EKPL removal, mean refractive power of the cornea (Km) revealed a tendency to increase from 43.8 D ± 3.4 (range, 36.62–50.75, *n* = 9) at pre-removal to 46.6 D ± 6.1 (range, 33.15–57.5, *n* = 10) at 6 months post-removal. The average amount of change in Km before and after removal was − 5.82 D ± 3.72 (range, 0.43–12.8 D). Out of nine cases, six cases showed corneal steepening and three cases revealed corneal flattening.

Contrary to prediction, corneal flattening was observed in two cases (Cases 8 and 10) who underwent EKP for high myopia correction.

To analyze whether the curvature of the cornea could restore the initial curvature after EKP removal, keratometric readings of pre-epikeratoplasty and post-lenticular removal were compared. Km was 44.6 D ± 1.0 (*n* = 8) preoperatively, and 47.1 D ± 6.8 (n = 8) at 6 month after EKPL removal.

When the keratometric readings of pre-epikeratoplasty and post-lenticular removal were paired and compared within the same case, the average difference was 5.1 D ± 4.0 (n = 8).

**Fig. 1:**
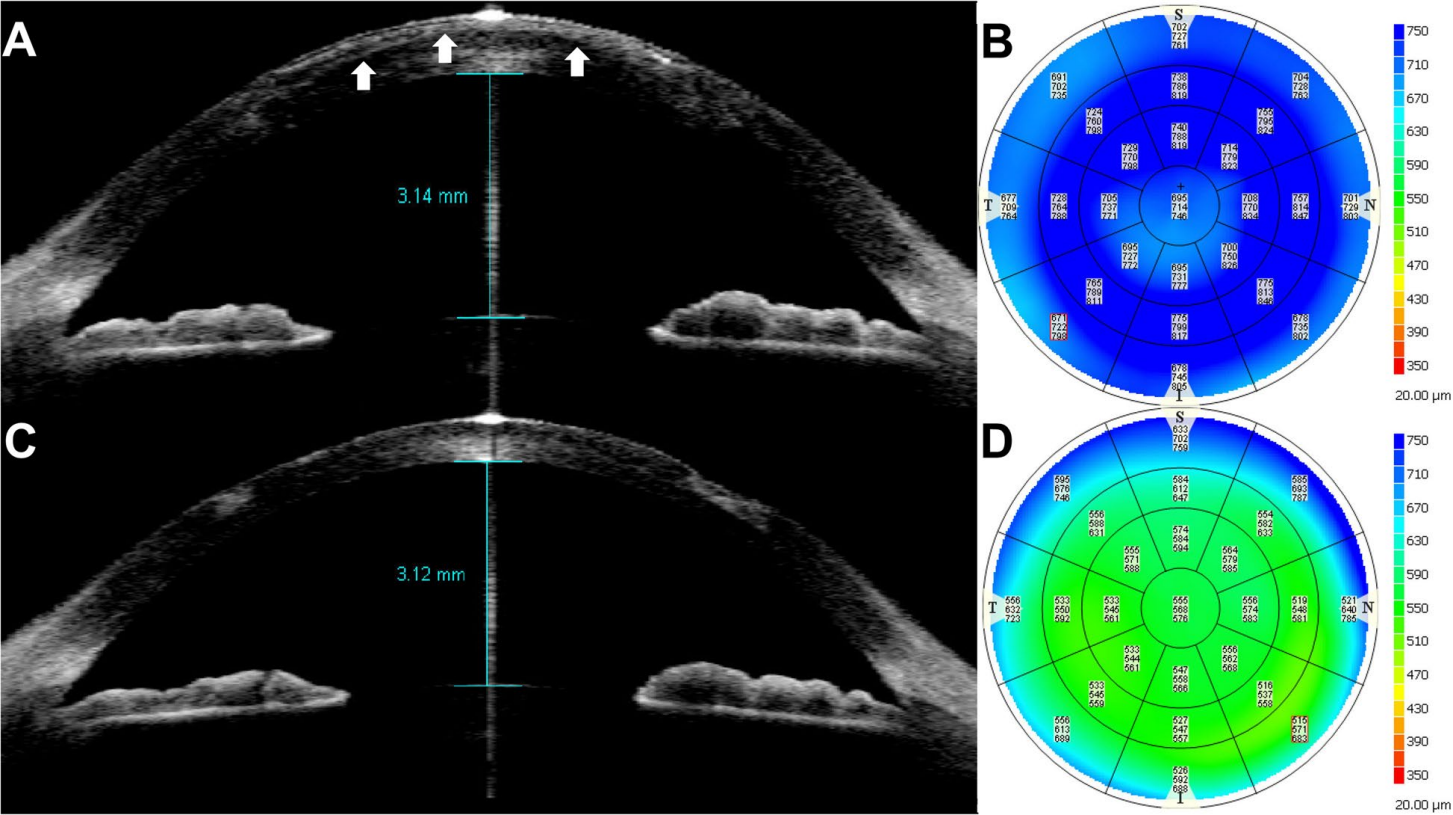
Horizontal scan of anterior segment optical coherence tomography (AS-OCT, A,C) and pachymetric maps measured by AS-OCT (**B**,**D**) in Case 10. Pre-removal AS-OCT (**A**) showed remarkable corneal opacity (white arrows). One month after lenticular removal, a significant reduction in corneal haziness was confirmed by AS-OCT (**C**), and corneal thickness had significantly decreased from 714 μm (**B**) to 568 μm (D)

All five patients who underwent lenticular removal due to graft opacity showed reduced corneal opacity (Fig. 1).

### Topographic changes after removal of lenticule

Table 3 presents an analysis of the topographies of six cases’ taken at pre-removal, and at one, six and twelve months after removal.

**Table 3:** Topography changes and complications after removal of lenticule

No	Sim K astig (D, degree)			Mean (D)			IRA in 3 mm (D)			CT		Comp
	BR	PR	FU	BR	PR	FU	BR	PR	FU	BR	PR	
4	4.4 × 83	N/A	8.8 × 85	47.3	N/A	33.1	±5.0	N/A	±7.2	339	292	Flattening
5	17.2 × 2	8.1 × 33	4.8 × 31	46.6	52.5	54.1	±11.3	±6.1	±6.0	719	566	
7	7.8 × 7	2.8 × 178	7.0 × 5	55.3	58.3	57.2	±6.9	±5.7	±10.8	733	573	Ectasia
8	2.4 × 159	1.8 × 91	1.4 × 101	45.3	46.0	45.9	±8.4	±3.0	±2.5	830	554	
9	1.1 × 82	2.4 × 91	1.7 × 75	40.3	45.0	44.7	±3.5	±2.9	±1.4	812	545	
10	1.5 × 28	1.9 × 85	3.2 × 117	48.1	49.0	47.2	±4.1	±5.0	±5.2	714	549	Epithelial ingrowh

*Sim K astig* Simulated keratometry astigmatism, *IRA* Irregular astigmatism, *CT* Corneal thickness, *Comp* Complication, *BR* Before removal, *PR* 1 month after removal, *FU* 6–12 months after removal

**Fig. 2:**
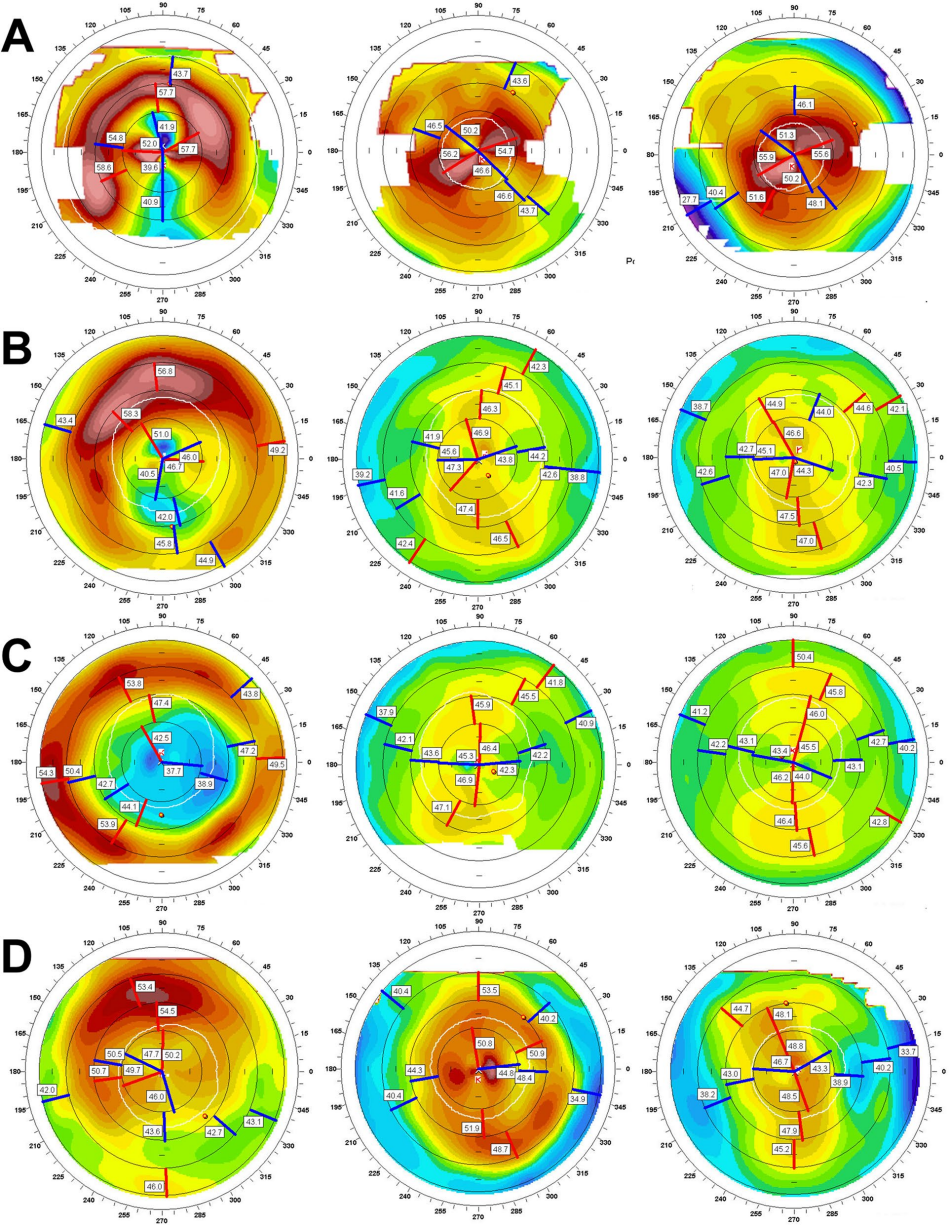
Corneal topographic changes before (first column), 1 month (middle column), and 6–12 months (last column) after the lenticular removal in case 5 (**A**), case 8 (**B**), case 9 (**C**) and case 10 (**D**). In cases 5, 8, 9, the cornea became steep after removal surgery, but in case 10, the cornea became slightly flat

**Fig. 3:**
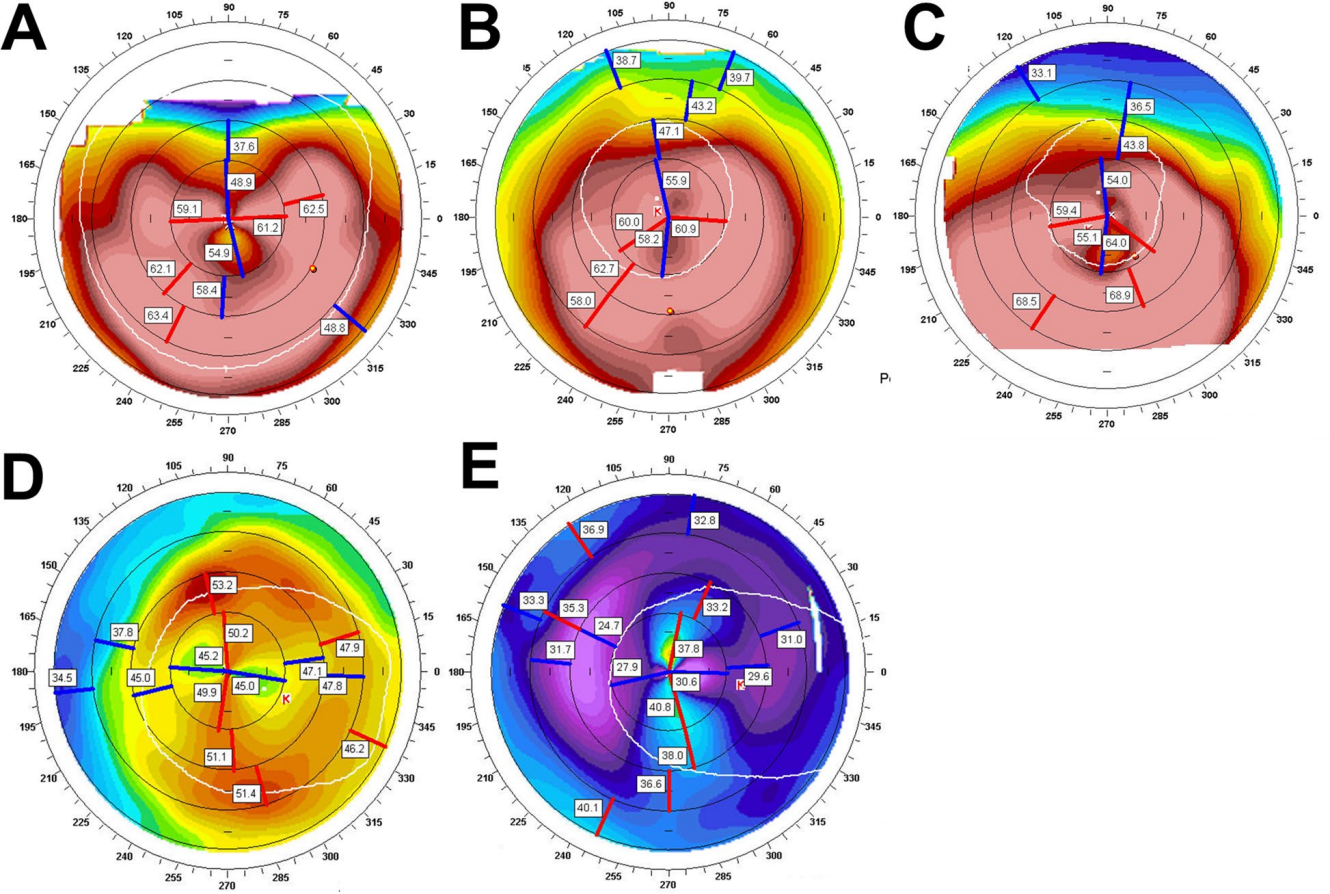
Aberrant corneal topography after removal of the lenticules. Corneal topography in case 7 at 21 years after epikeratophakia (**A**), 1 month after lenticule removal (**B**), and 2 years later (**C**). The axial map reveals rapidly increasing irregular astigmatism and corneal steepening, likely keratoconus. Corneal topography in case 4, 13 years after epikeratophakia (**D**), 7 months after lenticule removal (**E**). The axial map revealed an extremely flat cornea. The cornea became flatter than that before the epikeratoplasty

Simulated keratometry astigmatism (Sim K astig) increased in three cases after EKPL removal and decreased in the other three cases. Km increased in four cases (Fig. 2A-C, Fig. 3A-C) and decreased in the other two cases (Fig. 2D, Fig. 3D-E).

Depending on the different types of refractive errors to be corrected, the lenticule is shaped as a plus lens for aphakic hyperopia or a minus lens for myopia. Thus, in aphakic patients, the central cornea steepened in curvature by a lenticule that is thickest in the centre, and the central cornea is flattened in high myopic patients.

Therefore, the central cornea is supposed to flatten in patients with aphakia (Fig. 3D-E) and steepen in high myopia (Fig. 2A, C, Fig. 3A-C) after lenticule removal. However, the central cornea steepened in the two high myopic cases (Fig. 2B, D). These ectatic changes may be caused by the abnormal tensile strength of the collagen fibrils in high myopia [11].

When the Km at 1 month postoperatively and the Km after 6 months or more postoperatively were compared, they showed a relatively similar values (1 month vs 6–12 months; 50.2 D ± 4.84 vs 49.8 D ± 4.92, *n* = 5). Center corneal irregular astigmatism (IRA) within 3 mm also showed little change over time after removal, except for case 7, who developed keratoectasia (1 month vs. 6–12 months; 4.25 D ± 1.4 vs. 4.45 D ± 1.31, *n* = 4).

Compared to pre-removal data, IRA within 3 mm increased in three cases (pre-removal vs. 6–12 months post-removal; 5.5 D ± 1.4 vs. 8.0 D ± 2.8, *n* = 3), and decreased in the other three cases (pre-removal vs. 6–12 months post-removal; 7.73 D ± 3.2 vs. 3.3 D ± 2.0, n = 3). The central corneal thickness decreased significantly from 707 μm ± 156.5 to 519 μm ± 93.3 after EKPL removal (*n* = 6).

### Additional adverse event after removal of lenticule

**Fig. 4:**
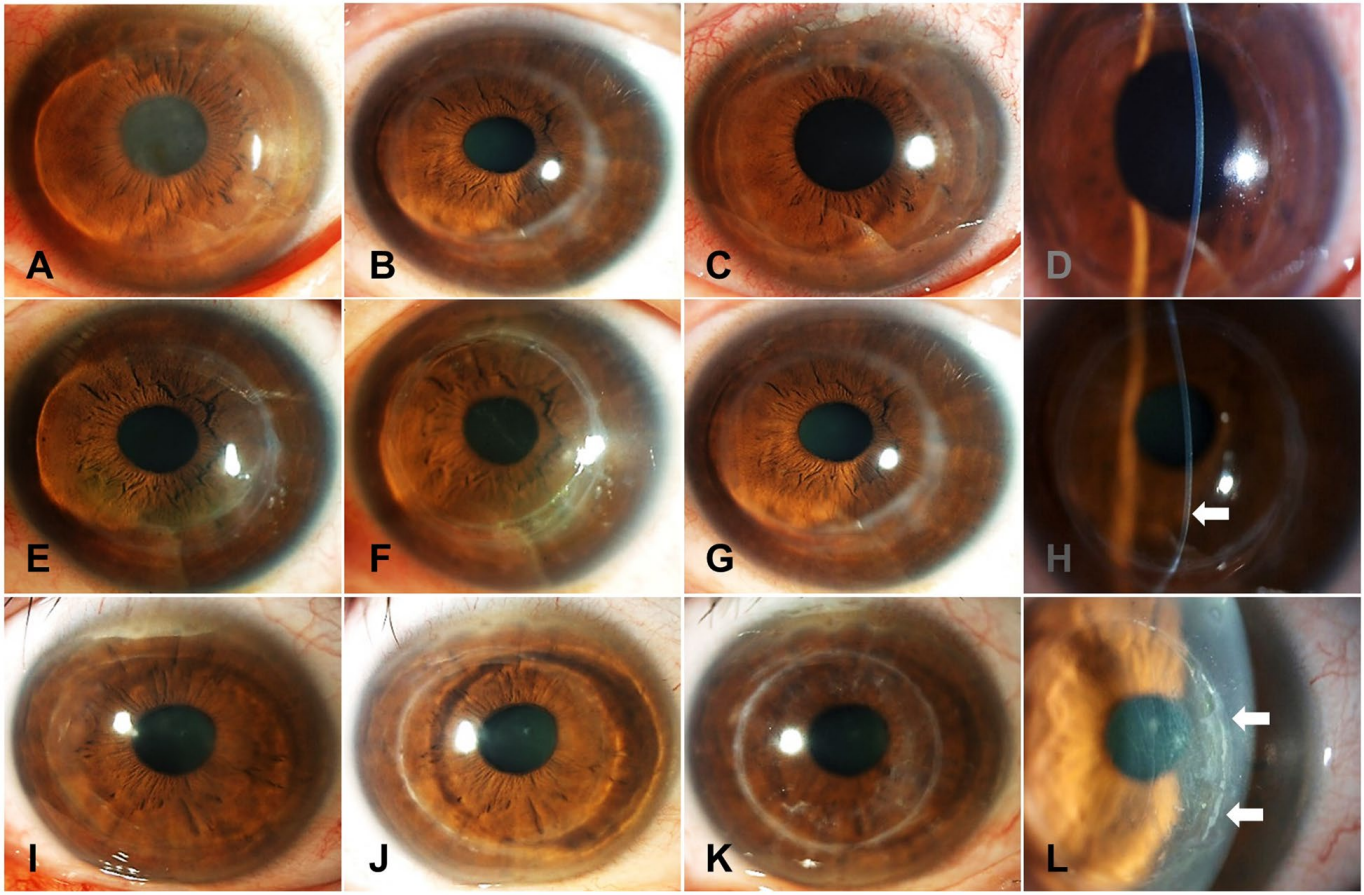
Representative photos of cornea before and after epikeratophakic removal. Photos of case 8 at 30 year follow-up after epikeratophakia before (**A**), 1 month (**B**), and 1 year (**C**) after lenticule removal. No opacity was observed with a normal thin cornea at 1 month after lenticule removal (**D**). Photos of case 7 at the year follow-up after epikeratophakia before (**E**), 1 month (**F**), and 8 years (**G**) after lenticule removal. Inferocentral corneal thinning and steepening were noted 8 years after lenticule removal (**H**). Photos of case 10 in 29 years after epikeratophakia, (**I**), 2 months (**J**), and 10 months after lenticule removal (**K**). Abnormal epithelial ingrowth was observed 2 months after the removal procedure (**L**)

Seven cases revealed a normal recovery process after removal without adverse effects (Fig. 4A-D), but complications were observed in 3 of 10 cases.

As post-lenticular removal complications, excessive corneal flatness was observed in case 4 (Fig. 3D, E), and corneal ectatic changes were observed in case 7 (Fig. 3A-C). Likely keratoconus, inferior corneal thinning, and protrusion were clearly visible on slit-lamp examination (Fig. 4E-H).

In case 10, the epithelial cells abnormally invaded the groove to the corneal surface and filled the gap with ingrowth of the epithelial cells at 2 months after the removal surgery (Fig. 4I-L). Abnormal epithelial ingrowth induced corneal deterioration, including opacity, irregular astigmatism, and abnormal photophobia symptoms (Fig. 4L). Therefore, the epithelial ingrowth was removed, and the corneal gap was sutured to prevent recurrence of epithelial ingrowth. The cornea remained stable without recurrence, and the photophobia disappeared (Fig. 4K).

## Discussion

To the best of our knowledge, this is the first descriptive study with the largest sample size to report postoperative complications and topography alterations in patients with EKPL removal.

To transplant the lenticule, the prepared lenticule was anchored to a groove made with a trephine. The lenticule was sutured in place with superficial sutures that were removed at approximately 3 weeks. An implanted lenticule is supposed to have an appropriate refractive power to correct the underlying refractive error. However, EKP is no longer performed because of its unpredictability and complications associated with the lenticule. After a long period of time has passed, patients sometimes return to the hospital requesting removal. In particular, in the case of patients requiring cataract surgery, EKPLs must be removed to determine the accurate power of the IOL. To inform the patient about the adverse events before the removal, we need to predict what clinical manifestations may appear after EKPL removal, and to know what side effects may occur.

Epikeratoplasty has potential complications, and reports have been published on failure to re-epithelialize, irregular astigmatism, graft haze, infection, and progressive myopia [9, 12–15]. Among our cases, opacity was observed in five cases, irregular astigmatism was revealed in one case, and severe myopic shift was present in three cases. A great myopic shift was observed in patients with high myopia who were corrected by EKP (Cases 2, 7, and 10). One aphakic case also revealed slight myopic alteration. It is not clear whether myopic progression is caused by a change in axial length or a change in the refractive power of the cornea or lens. However, considering that the patient was administered in their 20s and 30s for EKP, the possibility of axial length elongation is low. Therefore, it is reasonable to assume that corneal refractive power increases with a decrease in corneal tensile strength due to mid-peripheral circumferential cutting. The other possibility is that host corneal curvature, which has been altered by lenticule-pressing, may have lost its tension or denatured for some reason [10, 16]. The increased K value was confirmed in two cases (cases 7 and 10). Uusitalo et al. also described patients treated for pediatric aphakia by epikeratophakia with a follow-up of 3 to 5 years. During a span of 4 years, a mean myopic shift of − 0.40 D was documented. A myopic shift occurred in 30.2% of the eyes and a hyperopic shift in 9.4% [4].

Greenbaum et al. found the reversibility of the cornea after epikeratophakic removal in three cases [9] (Table 4). However, most studies presented that cornea was not reversible with a removal of epikeratophakia (Table 4). Bleckmann et al. reported that fixation of the epikeratophakia lenticules led to a 2 to 3.5 D reduction in the K value after the removal of the corneal transplants in two cases (one high myopic eye after 13 years, the other aphakia eye after 15 years), and Shin YJ et al. also reported that EPK led to an increase in corneal refractive power in three myopia cases (Table 4) [10, 16]. It indicates that changes of keratometric value may affect the power calculation of IOL when the cataract surgery is planned.

**Table 4:** Overview of previously published studies on epikeratophakia removal

Author	No.	Diagnosis	Reason for removal	Reversiblity	Mean FU (years)	Comp,%
Greenbaum [9]	3	Myopia, Aphakia	Irregular astigmatism Opacity, cataract surgery	o	11	0
Bleckmann [10]	2	Myopia, Aphakia	Opacity, cataract surgery	X	14	0
Shin YJ [5]	3	Myopia	Myopic regression	X	4.2	0
Present study	10	Myopia, Aphakia	Irregular astigmatism Opacity, cataract surgery	X	18.7	30

*No* Number of cases, *FU* Follow up, *Comp* Complication

In this study, there was a difference of 5.1 D ± 4.0 (*n* = 8), when comparing the initial cornea and the post-removal cornea in the same case. Although statistical analysis could not be done due to the small number of patients, 5.1 D was significant difference. Through this study, we suggest that the cornea can lose its reversibility, and both increase or decrease is possible after EKPL removal. Seven of the eight cases showed an increase in the K value, and only one case (No. 4, aphakia patient) showed a reduction in the K value. The reason why the cornea loses its reversibility and the K value change pattern is unpredictable cannot be proved in this study. However, it is speculated that this may be due to the interaction of the original cornea with the lenticule, decrease in recipient corneal tensile strength, and fibrosis in the process of wound healing, causing the cornea to be distorted [8, 10, 17]. In addition, there is a possibility that corneal stromal necrosis was revealed partially in the process of pressing the cornea with a lenticule for a long period of time, resulting in a change.

Shin YJ et al. suggested that follow-up of more than 6 months is necessary until the cornea is stabilized when considering lens extraction [16]. Given that the differences in Km ranged from 0.5 D to 2.25 D (mean 1.06 ± 1.0 D) between 1 and 6 months after lenticule removal, IOL power calculation should be considered at least 6 months later to reduce miscalculation..

Through topographic analysis before and after removal, we were able to identify changes that were not possible in keratometry, such as axial map, irregular astigmatism, and diagnosis keratoectasia in case 7 [18]. All irregular astigmatism in the topography was outside the normal range. This suggests that scarring of a recipient cornea after epikeratophakia or persistent groove after removal of the lenticule can cause deterioration of vision because of an irregular corneal surface [17, 19].

Here, we report three complicated cases associated with lenticular removal. In the previous studies, there were no reports of complications that occurred after removal; Greenbaum et al. reported three EKPLs removal and Bleckmann et al. also reported two EKPLs removal; however, both studies did not mention postoperative complication [9, 10].

As shown in Fig. 3A-C, rapidly progressive keratoectasia is observed in corneal topography. Whether this patient had innate keratoconus or if ectatic changes were complicated after lenticular removal could not be accurately discriminated because there was no initial topography of the cornea.

However, there is a possibility that corneal connective tissue disorder had occurred by digging a groove around the mid-peripheral cornea and spending a long time with the lenticle fixed to the groove. When observing the rapid progression as the lenticule was removed, it is possible that the lenticule mechanically pressed the cornea like a hard contact lens, and the progression was slowed during that time.

The abnormal corneal flattening change in case 4 may also have occurred due to an abnormality in connective tissue, caused by one procedure performed during EKP.

The abnormal epithelial cell proliferation seen in case 10 suggests the possibility that the wound healing process after EKP removal is different from that of the normal cornea, which lets the stromal gap persistently open to allow epithelial cell ingrowth.

To reduce the rate of corneal complications such as keratectatic changes or epithelial ingrowth into the previous pocket after lenticule removal, possible risk factors of the host cornea or some of the surgical tips during the removal should be noted. Given that the cornea is a 360-degree trephined to make the pocket for insertion of the lenticule wing, the anterior stromal collagens may no longer contribute to maintaining the tensile strength of the cornea. The trephine thickness of EKP is usually 180 μm [6]. Considering that post-LASIK ectasia can occur in percent tissue thickness alteration ≥40% or a low residual stromal bed (≤ 300 μm), [20] thin cornea (≤ 480 μm) or high myopia that may have a weak tensile strength of the collagen fibrils would be risk factors for post-ectatic changes. During removal of the lenticule, the boundary of the lenticule can be identified by pressing the cornea near the lenticule. Sinskey-hooking can be used to dissect the junction between the lenticule and the host cornea without further stromal damage near the junction. Thereafter, the peripheral edge of the lenticule can be easily lifted off with hard grasping of the lenticule using tooth-forceps. If the groove of the previous pocket is well aligned and closed, a therapeutic bandage contact lens can be applied without further procedures. If the groove is open and gapped, the groove should be closed by suturing to prevent epithelial cells from invading the groove.

Epithelial ingrowth can also occur after radial keratotomy or LASIK. RK involves a vertical incision, whereas both LASIK and EKP require a sloping incision. Corneal epithelial ingrowth associated with RK is rarely reported through perforation due to surgery or trauma. Therefore, epithelial ingrowth is observed on the endothelial surface in the perforation [21]. Post-LASIK epithelial ingrowth is frequently reported at the interface between the flap and stromal bed following LASIK, especially when the flap is lifted for retreatment [22, 23]. Like LASIK, the epikeratophakic procedure makes sloping pockets, and epithelial cells can easily invade through the sloping stromal gap. Therefore, it would be better to temporarily suture the gap when the groove is open and exposed.

In EKP, epithelial cells could be proliferated in stromal gap within groove. As for the removal method, mechanical debridement could be performed in the same way as Post-Lasik epithelial ingrowth, and it would be better to temporarily maintain an additional tightening suture to prevent recurrence.

This study has several limitations. Due to the limitations of the retrospective design of this study, it was difficult to collect all data, especially in the 90s. In addition, it failed to show statistical significance because the number of patients was small, and the range of change was small. Furthermore, histological analysis was not performed on the removed lenticule. Therefore, further studies that include histologic evaluation are necessary to reveal the cause of the complication accurately.

## Conclusions

In summary, if EKPL removal is needed for any reason, the surgeon should expect that the corneal refractive power may steepen or flatten in some cases with abnormal astigmatism and irregularity. Epikeratophakic eyes may exhibit serious ectatic changes and abnormal epithelial cell ingrowth by 30% after removal of epikeratophakic lenticules.

## Data Availability

All data generated or analysed during this study are included in this published article.

## Abbreviations

EKP: epikeratophakia
EKPL: epikeratophakia lenticular
PRK: photorefractive keratectomy
LASIK: laser in situ keratomileusis
LASEK: laser epithelial keratomileusis
SMILE: small incision lenticule extraction
M: male
F: female
SE: spherical equivalent refraction
BCVA: Best corrected visaul acuity
Km: mean refractive power of the cornea
D: diopter
Pre-EK: pre-epikeratoplasty
Post-EK: post--epikeratoplasty
BR: before lenticule removal
PR: post lenticule removal
N/A: not available
Sim K astig: Simulated keratometry astigmatism
IRA: irregular astigmatism
CT: corneal thickness
Comp: complication
IOL: intraocular lens
OCT: optical coherence tomography
